# Book and Pamphlet Notices

**Published:** 1859-10

**Authors:** 


### BOOK AND PAMPHLET NOTICES.

A Practical Treatise on Enteric Fever; its Diagnosis and Treatment: being an Analysis of One Hundred and Thirty Consecutive Cases, derived from Private Practice, and embracing a Partial History of the Disease in Virginia. By James E. Reeves, M. D. Philadelphia: J. B. Lippincott & Co. 1859.

On finding we had a new work on fever to notice, we acknowledge we anticipated anything but pleasure in the task, supposing we should have some new theory of the disease to encounter, just as unsatisfactory and as fanciful as others we have hitherto had to animadvert on. We find, however, on perusal, all cause for such apprehension to be groundless. The author, wisely eschewing speculation, plunges at once *in medias res*, and proceeds to a practical consideration of his subject.

Dr. Reeves *in limine* objects to the term "typhoid," as applied to the form of fever in which we so frequently meet with a lesion of the lower fifth of the small intestines, and prefers that of "enteric," as being more precise, and essentially characterizing the disease. Now, it must be remembered that this lesion of the intestine which gives so much weight to the proposal of substituting the latter prefix, is not one universally present in the fever in question, and cannot therefore be looked on as an essential symptom; besides, it exists in other diseases without being necessarily accompanied by typhoid symptoms, and even in typhoid fever itself the severity of the disease is not always in proportion to the local affection. We are therefore disposed to retain the proscribed term until we get a better. In our present state of knowledge it has one advantage—it involves no theory.

That it is very rare to meet that remarkable condition of Peyer's and Brunner's follicles in other acute diseases, we readily concede; yet Peyer's glands have been seen both raised and red in scarlatina by Louis, and Brunner's follicles more frequently still have been similarly affected in the same disease. We allow this lesion to be a very general concomitant of typhoid fever; but if we can show a single case presenting the same symptoms and observing the same progress of this fever, and yet on a post-mortem examination failing to manifest either an erythematous, an exanthematous or an ulcerated state of the ilium, we shall be justified in not considering the intestinal lesion as essential to typhoid fever. We can, in corroboration of our statement, with confidence, refer to the reports of the government fever hospitals of the United Kingdom, particularly those of Ireland, where this fever never ceases to exist,—where it, in fact, seems to assume a fixedness of endemic character.

The author proposes a division of the symptomatology of of "enteric" fever into three distinct forms: simple or mild, intermediate and malignant. This conduces to facility of description, and aids the student in arranging and comprehending the several phenomena thus systematically presented to his mind.

In the second chapter, we have a clear and faithful statement of the symptoms that usher in the affection, and of those which develope themselves during its progress. As the author proceeds, he dwells on the absolute and relative importance that attaches to each in diagnosis and prognosis. And here, in passing, we must say, that though our experience warrants us in sustaining his several statements in connection with this part of his subject, still we do not hesitate to state it would have, with advantage, admitted of further amplification. Every one who has seen practice, must be aware how valuable in fever the pulse is, in view to prognosis; yet here, about forty lines, and no more, are devoted to its consideration. There is no allusion to the abnormally slow or even the normal pulse of health occasionally observed in fever—both of serious augury. The slow pulse of convalescence and the other varieties of pulse with which practical men are familiar, are altogether unnoticed. The same observations will apply to every other symptom considered with a view to prognosis. Dr. Reeves has limited himself to a space within which he cannot profitably comprise so many and such important objects of enquiry. The chapter on "Anatomical Lesions" is simply correct, but adds nothing to what we learned long since from the French pathologists. Much pains seem to have been taken in collecting the opinions of his medical friends respecting their views generally of the disease and their experience of its first advent to their respective districts. The replies to the author for the most part imply considerable intelligence and close observation of disease, both of which, from our personal knowledge of the profession in Virginia, we were quite prepared to expect. Our space will not permit us to follow Dr. Reeves whilst engaged in the task of searching out the causes, determining the duration and unravelling the complications of typhoid fever. We must hasten on to that part of the work wherein the treatment is considered.

The author advocates the employment of emetics at an early stage of the disease, whenever there is nausea and a bilious coating on the tongue. Sometimes, even at an advanced stage, he thinks, under certain circumstances, they may be useful. Though, under certain circumstances, the treatment of fever may with advantage be commenced with emetics, yet the administration of an emetic may be followed by the worst consequences—if there be tenderness of the epigastric region, or a tongue indicating any amount of gastritis, an emetic most probably may manufacture much trouble for the physician and danger for his patient. How often have we seen the tongue become dry and red, tympanitis rapidly set in and other grave symptoms quickly follow the ill-timed and otherwise injudicious administration of an emetic?

In the commencement of the volume the author very properly animadverts on the impropriety of increasing the irritability of the bowels by vain efforts "to purge off" what patients often considered a bad cold—yet under the head of treatment we find him approving of the administration in the early stage of a brisk purgative of calomel and jalap. This does not quite accord with our notion of safe practice. Two more dangerous medicines in typhoid fever cannot be employed. Many a disastrous termination can be traced to the employment, even at an early stage, of "a brisk purgative." We well remember the days of calomel and black draught—the deadly weapons with which each medical onslaught was commenced—with such fatal effect to unfortunate patients, "pollas d'ipthimous psuchas Aidi proiapsen."

Dr. Reeves next considers the expediency of giving small doses of mercury—"When the tongue is dry, the skin parched, scanty urine, diarrhœa and tympanitis, delirium or stupor, twitchings of the tendons, a small and weak pulse, the propriety of small doses of mercury, with a view to its specific effect, by some authorities, is urged with no little emphasis." He refers to Dr. Wood as an authority in support of its exhibition. This was the fashionable practice some twenty years ago or more, at the other side of the Atlantic, and worse could not be well conceived. It succeeded the mania in the profession for phlebotomizing, which was about the most murderous means medical ingenuity ever devised. If the former slew its thousands, the latter slew its tens of thousands.

"General blood-letting is now very rarely practised in enteric fever. Formerly the same caution and nice discernment now observed in its employment was not exercised, and to this, more than from any other cause, perhaps, may be attributed the very great increase in the number of recoveries in late years. In those cases which bear a severe character from the beginning, where headache is violent, increased sensibility to light and sound, the pulse full and strong, high fever, with a robust and plethoric habit, it may be proper to abstract a moderate quantity of blood from the arm; but unless it is resorted to during the first two or three days of the disease, it had better be omitted." This is eminently practical and exactly reflects our own opinion.

Veratrum viride, as a remedy in fever, next receives the author's attention.

When we consider that in fever the primary symptoms mark a depressed and enfeebled state of the nervous system, we question much the prudence that would suggest the administration of a medicine like veratum viride, the action of which must either directly or indirectly increase the very state it is our aim to combat. Individually, we have no experience of the medicine in this disease, but nothing short of evidence, resulting from the extensive and careful observation of numerous cases, suitably selected to test the matter, can reconcile us to its employment in a disease where its properties appear so little in accordance with the indications we have to fulfil. We regret our space will not permit us to pass in review the various other remedial agents advocated by the author. His views in reference to oil of turpentine, opium and astringents seem in the main correct. We think he might have dwelt with advantage at greater length on the complications so often to be encountered in typhoid fever. He certainly entertains a most profound respect for the opinions of Dr. Wood, whom he seems to consider the grand master, but he could have profitably extended his enquiries further and learned what others had done and are doing in the same field elsewhere. In a treatise like this we should expect that the labors of those who have been amongst the most distinguished of our profession, and whose opportunities for observation were most extensive, should not have been ignored.

This disease is one of those most common in the practice of the profession at the other side of the Atlantic, and one to which the attention of the profession in Britain, Ireland and France has been for many years most unremittingly directed; how strange then that a work specially devoted to this subject should be limited in the materials of its construction to a sphere of observation confessedly narrow and comparatively sterile! For the very accurately detailed anatomical lesions of the fourth chapter we are more indebted to the French School than to Dr. Wood, the object of our author's professional idolatry. Still, though we may not find this book altogether faultless, and in this it but partakes of what is incidental to all human productions, yet on the whole it is a practical and truthful volume—evidently written by one who loves his profession and has a practical knowledge of the subject. It is a book well calculated for the student, who can at once enter upon the study of the disease without having his mind confused by those learned yet unprofitable speculations that serve so frequently as a preface to works professing to treat of typhoid fever.

We acknowledge the receipt of the following books from W. B. Keen, 148 Lake street, and, excepting one already referred to, shall notice them more fully in next No. of the Journal:

The Action of Medicines in the System; or, "On the Mode in which Therapeutic Agents introduced into the Stomach produce their peculiar effects on the Animal Economy." Being the Prize Essay to which the Medical Society of London awarded the Fothergillian Gold Medal for 1852. By Frederick William Headland, M.D., B.A., F.L.S., Licentiate of the Royal College of Physicians, etc., etc. Third edition, revised and enlarged. Philadelphia: Lindsay & Blakiston. 1859.

Alcohol: Its Place and Power. By James Miller, Professor of Surgery in the University of Edinburgh, Surgeon in Ordinary to the Queen for Scotland, etc., etc. From the nineteenth Glasgow edition. Philadelphia: Lindsay & Blakiston. 1859.

The Use and Abuse of Tobacco. By John Lizars, late Professor of Surgery to the Royal College of Surgeons, and lately Senior Operating Surgeon to the Royal Infirmary of Edinburgh. From the eighth Edinburgh edition. Philadelphia: Lindsay & Blakiston. 1859.

The Physician's Visiting List, Diary, and Book of Engagements, for 1860. Philadelphia: Lindsay & Blakiston.

---

THE DENTAL COSMOS.

We have received the first number of *The Dental Cosmos*, published by Jones & White, of Philadelphia, and edited by J. D. White, M.D., D.D.S., J. H. McQuillen, D.D.S., and Geo. J. Zeiglen, M.D.

The "*Cosmos*" takes the place of the "*Dental News Letter*," (quarterly,) and is to be a monthly publication; each number contains fifty-six pages. It takes a first rank among dental publications.

